# Smartphone-based screening for visual impairment in Kenyan school children: a cluster randomised controlled trial

**DOI:** 10.1016/S2214-109X(18)30244-4

**Published:** 2018-07-14

**Authors:** Hillary K Rono, Andrew Bastawrous, David Macleod, Emmanuel Wanjala, GianLuca DiTanna, Helen A Weiss, Matthew J Burton

**Affiliations:** a International Centre for Eye Health, Clinical Research Department, London School of Hygiene & Tropical Medicine, London, UK; b Kitale County and Referral Hospital, Kitale, Kenya; c The Peek Vision Foundation, London, UK; d Medical Research Council Tropical Epidemiology Group, London School of Hygiene & Tropical Medicine, London, UK; e Cornea and External Eye Department, Moorfields Eye Hospital NHS Trust, London, UK

## Abstract

**Background:**

Childhood visual impairment is a major public health concern that requires effective screening and early intervention. We investigated the effectiveness of Peek school eye health, a smartphone-based sight test and referral system (comprising Peek Acuity test, sight simulation referral cards, and short message service [SMS] reminders), versus standard care (Snellen's Tumbling-E card and written referral).

**Methods:**

We initially compared the performance of both the Snellen Tumbling-E card and the Peek Acuity test to a standard backlit EDTRS LogMAR visual acuity test chart. We did a cluster randomised controlled trial to compare the Peek school eye health system with standard school screening care, delivered by school teachers. Schools in Trans Nzoia County, Kenya, were eligible if they did not have an active screening programme already in place. Schools were randomly allocated (1:1) to either the Peek school eye health screening and referral programmes (Peek group) or the standard care screening and referral programme (standard group). In both groups, teachers tested vision of children in years 1–8. Pupils with visual impairment (defined as vision less than 6/12 in either eye) were referred to hospital for treatment. Referred children from the standard group received a written hospital referral letter. Participants and their teachers in the Peek group were shown their simulated sight on a smartphone and given a printout of this simulation with the same hospital details as the standard referral letter to present to their parent or guardian. They also received regular SMS reminders to attend the hospital. The primary outcome was the proportion of referred children who reported to hospital within 8 weeks of referral. Primary analysis was by intention to treat, with the intervention effect estimated using odds ratios. This trial is registered with Pan African Clinical Trial Registry, number PACTR201503001049236.

**Findings:**

Sensitivity was similar for the Peek test and the standard test (77% [95% CI 64·8–86·5] *vs* 75% [63·1–85·2]). Specificity was lower for the Peek test than the standard test (91% [95% CI 89·3–92·1] *vs* 97·4% [96·6-98·1]). Trial recruitment occurred between March 2, 2015, and March 13, 2015. Of the 295 eligible public primary schools in Trans Nzoia County, 50 schools were randomly selected and assigned to either the Peek group (n=25) or the standard group (n=25). 10 579 children were assessed for visual impairment in the Peek group and 10 284 children in the standard group. Visual impairment was identified in 531 (5%) of 10 579 children in the Peek group and 366 (4%) of 10 284 children in the standard care group. The proportion of pupils identified as having visual impairment who attended their hospital referral was significantly higher in the Peek group (285 [54%] of 531) than in the standard group (82 [22%] of 366; odds ratio 7·35 [95% CI 3·49–15·47]; p<0·0001).

**Interpretation:**

The Peek school eye health system increased adherence to hospital referral for visual impairment assessment compared with the standard approach among school children. This indicates the potential of this technology package to improve uptake of services and provide real-time visibility of health service delivery to help target resources.

**Funding:**

Seeing is Believing, Operation Eyesight Universal, Queen Elizabeth Diamond Jubilee Trust, and Wellcome Trust.

## Introduction

Worldwide, an estimated 19 million children have visual impairment (defined as Snellen visual acuity of <6/12 [or <20/40] in the better-seeing eye). Visual impairment can have a profound effect on child development, quality of life, educational attainment, and economic productivity.^1^, ^2^ The leading cause of visual impairment in children is uncorrected refractive error, affecting approximately 12 million children, which can be easily corrected with spectacles.^3^ Many school children are held back by poor sight for lack of this simple intervention. Most children with visual impairment live in low-income countries.^4^ In Kenya, for example, the estimated prevalence of visual impairment among school children (6–20 years) ranges from 4·8% to 5·6%.^5^, ^6^ In Asian populations, estimates range from 6·4% to 22·3%.^7^, ^8^

Addressing childhood blindness and visual impairment is a major priority for VISION2020, a global programme fighting avoidable blindness led by WHO and the International Agency for Prevention of Blindness.^9^ To reduce childhood visual impairment, the programme promotes vision screening of all children who go to school and promotes integration of vision screening into school health programmes by 2020. Vision testing to identify children with correctable visual impairment enables interventions to be offered early, before educational and social progress is adversely affected.^10^

**Research in context**

**Evidence before this study**

A systematic review of mobile health (mHealth) applications for vision testing identified numerous available applications; however, very few had undergone validation or certification. mHealth systems have shown promise for improving health-care delivery although no trials of mHealth interventions to improve eye health have been published.

**Added value of this study**

This study showed both the feasibility of effective task-shifting to teachers using the Peek school eye health system to identify and refer children with sight problems and substantially increased adherence to referral (within 8 weeks of screening) of those identified by establishing a closed-loop between screeners (teachers) and the service provider (hospital).

**Implications of all the available evidence**

Poor vision has negative social, health, educational, and economic consequences. Early identification and treatment of eye conditions reduces the prevalence of visual impairment. Our results have shown that the Peek school eye health system, when used by teachers, is effective for identification and referral, as well as providing live health system data with evidence of barriers to service delivery. The lessons learned from this trial have been adopted and scaled up in Kenya by the Ministries of Health and Education to a countywide programme, serving 200 000 children. Additionally, this programme has been replicated and further developed in India and Botswana, which is taking it to a national scale.

Vision screening of children in Kenya is guided by school policies.^11^ In areas with active programmes, trained hospital-based clinical officers and ophthalmic nurses usually carry out the screening in schools. This procedure requires eye-care workers to leave their usual workplace (hospital eye clinics), thus reducing the availability of these services. In a pilot school-screening programme in Trans Nzoia County, Kenya, we trained school teachers to identify children with visual impairment using a Snellen Tumbling-E card. Children passed or failed at two predefined threshold levels: 6/60 (20/200) and 6/12 (20/40), in either eye. A hospital referral was made for children failing at either level by sending a letter to the child's parent or guardian explaining the need to access care. However, only a few children attended this hospital referral. Multiple barriers to care include communication failure between pupils or schools and parents or carers, as well as between schools or carers and hospitals, the inaccessibility of services, direct and indirect costs, myths related to treatment, and fear.^12^

Access to a connected mobile device in sub-Saharan Africa has increased dramatically in recent years, from 1% in 2002 to around 75% in 2016.^13^, ^14^ This increase in use is resulting in profound improvements in communication and commerce, and opens new opportunities for health care. Use of mobile health (mHealth) interventions to support communication between providers and patients through short messaging services (SMS) can promote access to health care.^15^ Previously, we developed and tested a smartphone application for Tumbling-E visual acuity testing (Peek Acuity app) to measure visual acuity in adults in Kenya. This test was accurate and repeatable, and acceptable to patients, examiners, and stakeholders.^16^, ^17^ We have now integrated this app into an mHealth system for vision screening among school children. The aims of this study were to validate the Peek school eye health system and to assess the effect of this system on the referral rate of children with visual impairment compared with the standard visual screening system currently used in Kenya.

## Methods

### Study design and participants

We first did a validation study to confirm that the teachers could be trained to carry out vision screening. We compared the performance of both the Snellen Tumbling-E card and the Peek Acuity test with a standard backlit EDTRS LogMAR Tumbling-E visual chart (Precision Vision, Woodstock, IL, USA) in measuring visual acuity in children. The order of the assessments was random. This validation study was carried out in three schools not involved in the subsequent trial.

We then did a single-masked, parallel-group, cluster randomised controlled trial in 50 primary schools in Trans Nzoia County, Kenya. Clusters were individual schools with no active visual screening programme in place. School children were tested for visual impairment by teachers who were trained to use either the standard school screening system Snellen Tumbling-E card and paper referral) or the Peek school eye health system. CONSORT guidelines for reporting cluster randomised trials were followed.^18^

All pupils attending years 1–8 in the selected schools were eligible for inclusion. Children were provided with information and consent forms to give to parents or guardians who were then requested to give written informed consent for teachers to test eye sight before enrolment. Children were excluded if they were unwilling or unable to give verbal consent, or if their parents or guardians did not provide consent.

The study was approved by the Moi University Institutional Research and Ethics Committee, Kenya and the London School of Hygiene & Tropical Medicine Ethics Committee, UK. Permission was also granted by Trans Nzoia Education and Health authorities, Kenya. The study adhered to the principles of the Declaration of Helsinki on Ethics.

### Randomisation and masking

Schools were randomly assigned (1:1) to either the Peek school eye health system (Peek group) or the standard school screening system (standard group). Geocoordinates of all eligible schools were obtained. To minimise imbalance in geographical location between the two groups, a statistician used a minimisation-based algorithm in R based on the geographical location (six zones, each covering 60 degrees of a circle around Kitale hospital) of the schools and their distance from the hospital, using random permuted blocks.^19^, ^20^ Using this balance algorithm, we obtained a set of optimal allocations and sampled the final distribution of allocations from this set of optimal allocations.

We could not mask the study team providing training, or mask participants and teachers to the screening method being used. The primary outcome data were collected by one hospital clerk who was masked to the screening method used. On arrival at the hospital, the child's parent or guardian presented a referral slip, which was identical for each group. Children who attended the hospital appointment in the Peek group were also marked as attended in the hospital app by a different clerk to those who received them at reception, to maintain masking of the primary outcome data collection.

### Procedures

We selected 25 teachers, who had previously been trained to use the standard system as part of the pilot school-screening programme, on the basis of their availability and activity during the pilot. We trained them for 1 week on how to operate a smartphone and how to screen and refer using both methods (Peek and standard). We allocated teachers and transported them to schools, where they did not work, in a manner that ensured a teacher screened at two schools each, one from each group. The teachers screened the children class by class. We classified children in years 1–3 as lower primary school and those in years 4–8 as upper primary school. We recorded age, sex, and education level for each child in the study. For those who screened positive (ie, could not see 6/12 in either eye), we collected additional information for contact and follow-up purposes: child's name, parent's name, primary language, and contact number.

**Figure 1:**
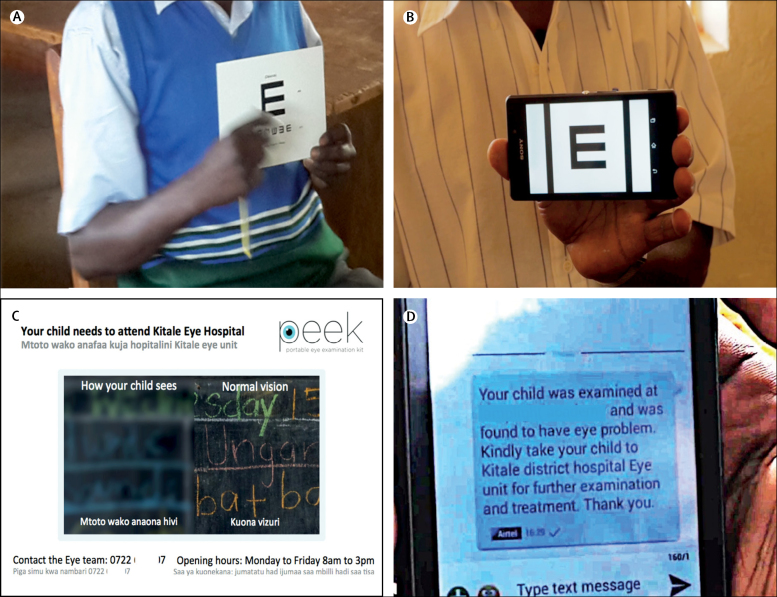
Vision screening methods used in school children (A) Standard screening with a Tumbling-E card. (B) Peek Acuity screening app used on a smartphone. (C) Peek referral card showing the vision of the child and the referral instructions. (D) Parent receiving an SMS message with instructions after screening. SMS=short message service.

In schools allocated to the standard group, the teacher tested the children's sight for each eye separately. The eye not being tested was covered with an occluder. The child was shown a Tumbling-E vision screening card (figure 1A) at a distance of 3 m. This card has a row of five letter Es, in four different orientations. The size of the letters at this test distance corresponds to a visual acuity of 6/12. The child passed the 6/12 threshold test if they correctly identified the direction of four of the five letter Es. If they failed at 6/12, they were shown the 6/60 card, which has larger letter Es, and again passed if four of five were correctly identified. The result was recorded for each eye separately as: can see 6/12, cannot see 6/12 but sees 6/60, or cannot see 6/60. Children who could not see 6/12 in either eye were referred to Kitale hospital. The paper referral form was completed in triplicate: one copy given to the child, advising the parent to take the child to hospital, one copy to the head teacher, and one copy sent directly to the hospital.

In schools allocated to the Peek school eye health system, the teacher used the Peek Acuity vision screening app on a smartphone (Samsung Galaxy S3) at 2 m. Each eye was tested separately, with the fellow eye covered with an occluder. A series of up to five Tumbling-E optotypes equivalent in size to Snellen 6/12 (20/40, LogMar 0.3) were presented randomly in one of four orientations (figure 1B). The child pointed in the direction they perceived the arms of the letter E to be pointing, and the teacher used the phone's touch screen to swipe in the same direction to enter the child's response, without looking at the phone's screen. One optotype was presented at a time. The test automatically concluded when the threshold number of passes (four of five) or fails (two of five) at the 6/12 optotype size was reached.^16^ If the child failed the 6/12 level, the app automatically presented a 6/60 sized optotype and the test was repeated to determine whether or not 6/60 (20/200, LogMAR 1.0) could be seen. At the end of the test, if the child failed the 6/12 level in either eye (ie, screened positive), the app prompted the collection of referral details (patient's or guardian's name, local language, and mobile phone number) and generated a referral to the hospital. A child who screened positive was given a printed referral photo card with their name, hospital contact details, and opening times to take home. The card included a split image with one half blurred to the same degree as the child's visual impairment (figure 1C). When connected to the internet, the app sends this referral details to a cloud-based server, which automatically generated a personalised SMS that was then sent to the child's parent or guardian with advice on the outcome of the eye assessment and instructions for referral in the chosen local language (figure 1D). A contact person (usually the head teacher for schools) also received an SMS with a list of children found to be visually impaired, needing referral. The messages were resent at intervals of 2 weeks until the child attended the hospital or for a maximum of 8 weeks. A referral was also automatically sent to the hospital where a database of referred children was kept accessible through a hospital reception app.

The follow-up period of this trial was 8 weeks. On presentation to the eye department at Kitale hospital for assessment, a clerk recorded the attendance of the referred child. The clinical team assessed the child to determine the level of vision, cause of visual impairment, and any treatment needed. Interventions included provision of eye drops, spectacles, or surgery. The team assessed visual acuity using a 6 m Snellen chart and classified the cause of vision loss on the basis of common treatable or preventable causes.^21^ All children with visual impairment received free treatment at hospital.

### Outcomes

The primary outcome, which was centrally assessed, was the proportion of referred participants who attended the Kitale hospital eye department within 8 weeks of referral. The main secondary outcome was the time taken by children with visual impairment to reach hospital. We also report the level of vision measured in hospital and the causes of visual impairment identified.

### Statistical analysis

We calculated the sample size assuming a visual impairment prevalence of 4·8% (<6/18 in better eye) and average school size of 542 pupils (about 25 visually impaired children per school).^5^ Assuming a design effect of 1·24 (ie, intraclass correlation coefficient 0·01) at least 21 schools were required in each group to provide 80% power to detect a difference of 10% (60% in the Peek group *vs* 50% in the standard group) in overall hospital attendance within 8 weeks. However, to ensure enough power would be retained to detect this difference if some schools dropped out of the study, we selected a final sample of 50 schools (25 in each group), providing 88% power to detect this difference if all schools participated.

For the initial validation study, we defined a child as visually impaired if they had at least one eye classed as having vision worse than 6/12 (or worse than 0·3 when using LogMAR). Using ETDRS LogMAR as the reference test, and the previous definition of visual impairment as the outcome, we estimated the sensitivity, specificity, positive predictive value, and negative predictive value for Peek and Tumbling-E cards.

The analysis was by intention to treat. For the primary outcome analysis, we used mixed effect logistic regression to estimate the odds ratio (OR), comparing the odds of attendance within 8 weeks of referral between the control (standard) and intervention (Peek) groups, first unadjusted and then, in case of any imbalance between demographics in the two groups, adjusted for age, sex, education level, and distance to hospital.

We generated Kaplan-Meier (K-M) survival curves to illustrate the difference in time-to-attendance between the two groups. We assessed the difference in time-to-attendance with hazard ratios (HRs) estimated by Cox regression, with a shared frailty at school level, first unadjusted and then adjusted for age, sex, education level, and distance to hospital. We checked Schoenfeld residuals and did a test of proportionality of hazards to identify if the assumption of proportional hazards was valid.^22^ In the case of the proportional hazards assumption being violated, we estimated HRs for narrower time bands, within which the proportional hazard assumption holds. We assessed the relationship between level of vision and diagnosis at hospital descriptively. We used STATA version 13 (STATA Corp, TX, USA) for the analysis.

The trial was registered with the Pan African Clinical Trial Registry, number PACTR201503001049236.

### Role of the funding source

The funders of the study had no role in the study design, data collection, data analysis, data interpretation, or writing of the report. The corresponding author had full access to all the data in the study and had final responsibility for the decision to submit for publication.

## Results

In the validation study, we tested the visual acuity of 1862 children using Peek Acuity, the standard Tumbling-E card, and ETDRS LogMAR (the reference test). Prevalence of visual impairment, as measured by ETDRS LogMAR (at least one eye with <6/12 vision), was 4% (n=65). Peek correctly identified 50 of 65 children as visually impaired (sensitivity 76·9% [95% CI 64·8–86·5]) and standard E-cards detected 49 of 65 children (sensitivity 75·4% [63·1-85·2]) (table 1). 12 (80%) of 15 children with visual impairment not identified by Peek had a LogMAR score in their worse eye of less than 0·3 and better than or equal to 0·4. With standard E cards, 15 (94%) of 16 children fell within this region of mild visual impairment, suggesting that it was mostly children with milder visual impairment that were missed by Peek and E cards. The specificity of Peek was lower (91%) than that of standard E cards (97%). Peek had a lower positive predictive value (23% [95% CI 17·7–29·4]) than the E card (52% [95% CI 41·1–62·0]) due to Peek's lower specificity (table 1).

**Table 1:** Performance of each test of visual impairment in the validation study

	Number of children who failed 6/12*test in at least one eye (N=1862)	**Sensitivity**	**Specificity**	**Positive predictive value**	**Negative predictive value**
LogMAR†	65 (4%)	..	..	..	..
Standard‡	95 (5%)	75·4% (63·1–85·2)	97·4% (96·6–98·1)	51·6% (41·1–62·0)	99·1% (98·5–99·5)
Peek‡	216 (12%)	76·9% (64·8–86·5)	90·8% (89·3–92·1)	23·1% (17·7–29·4)	99·1% (98·5–99·5)

Data are n (%) or % (95% CI).

* LogMAR value 0·3.

† Test done by ophthalmic clinical officer.

‡ Test done by teacher.

**Figure 2:**
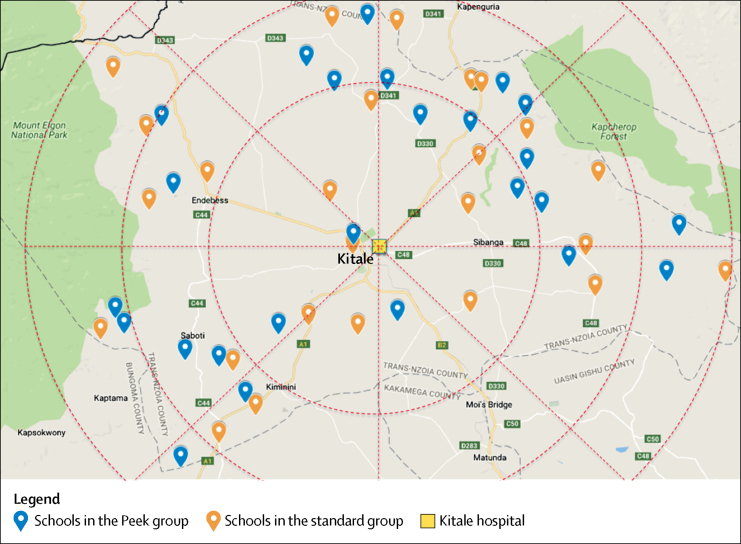
Location of primary schools in each study group in Trans Nzoia County, in relation to Kitale hospital, Kenya

**Figure 3:**
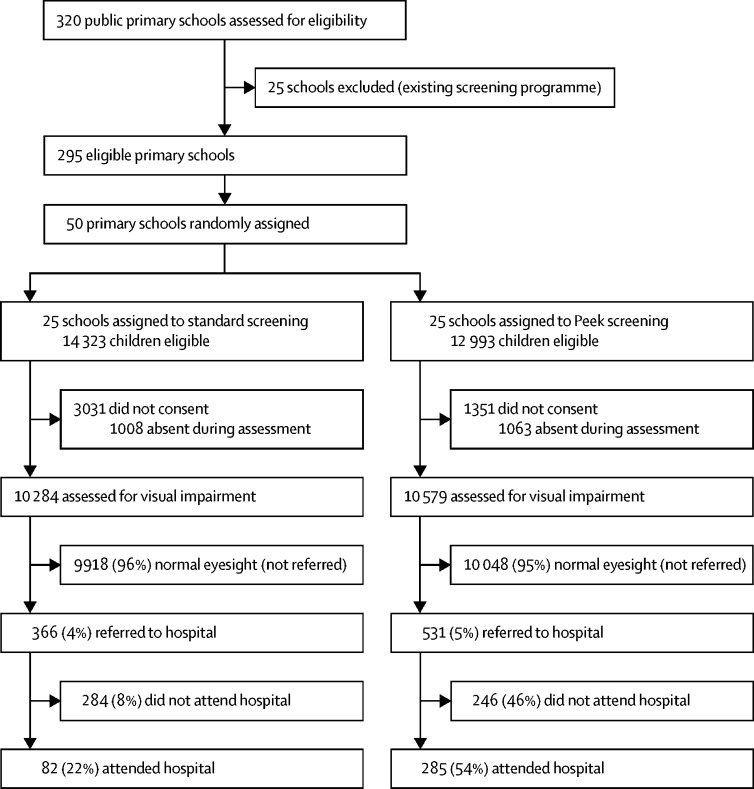
Trial profile

Trial recruitment occurred between March 2, 2015, and March 13, 2015. The final 8-week follow-up period finished on May 8, 2015. Of the 320 public primary schools, 25 were excluded as they already had active school screening programmes. Of the remaining 295 eligible schools, 50 were randomly selected and 25 were allocated to each group (figure 2). The mean distances between the schools in which screening took place and the hospital, and school sizes were similar in each group (table 2). All 27 316 potentially eligible children attending the 50 schools were invited for vision screening. Parental consent and child's assent were granted for 22 934 (84·0%) children (78·8% in the standard group and 89·6% in the Peek group), of whom 20 863 (91·0%) were assessed during a 2-week period (figure 3).

**Table 2:** Baseline characteristics of the schools and study participants

	**Peek group**	**Standard group**
Number of schools	25	25
Mean number of children per school, n (range)	423 (223–1135)	411 (270–1037)
Mean distance from school to Kitale hospital, km (range)	21·1 (1·9–50·6)	19·0 (1·8–37·6)
Number of children examined	10 579	10 284
Male sex	5303 (50%)	4953 (48%)
Mean age, years (SD)	11·2 (2·8)	11·4 (2·7)
Lower primary years 1–3	3744 (35%)	3236 (32%)
Upper primary years 4–8	6835 (65%)	7048 (69%)

Data are n (%), unless otherwise specified.

In this study, 531 (5%) of 10 579 children in the Peek group and 366 (4%) of 10 284 children in the standard group failed the screening test. Of these 897 referred children, 379 (42%) children were boys, with a mean age of 11·6 years (2·9), and 273 (30%) children were in lower primary; characteristics were similar between groups (table 3).

**Table 3:** Proportion of children with visual impairment and proportion who presented to hospital (primary outcome)

	**Peek group**	**Standard group**
Children with visual impairment on screening referred to hospital*		
Number of children	531 (5%)	366 (4%)
Male sex	226 (43%)	153 (42%)
Mean age, years (SD)	11·5 (3·0)	11·7 (2·8)
Lower primary years 1–3	179 (34%)	94 (26%)
Upper primary years 4–8	352 (66%)	272 (74%)
Children with visual impairment on screening who presented at hospital*		
Number of children	285 (54%)	82 (22%)
Male sex	130 (46%)	35 (43%)
Mean age, years (SD)	11·6 (2·9)	11·5 (2·6)
Lower primary years 1–3	88 (31%)	16 (20%)
Upper primary years 4–8	197 (69%)	66 (72%)
**Children who could not see 6/12 in either eye in hospital visual acuity test**		
Number of children	68 (25%) 276†	37 (47%) 78‡

Data are n (%), unless otherwise specified.

* Visual impairment defined as vision less than 6/12 in either eye.

† Vision from nine children was not recorded.

‡ Vision from four children was not recorded.

Of the 366 children referred from the standard group, 82 (22%) presented to the hospital during the 8-week follow-up period compared with 285 (54%) of 531 children referred from the Peek group. After adjusting for school clustering, children referred with the Peek school eye health system were more likely to attend hospital within 8 weeks than children referred with the standard screening system (OR 7·35 [95% CI 3·49–15·47]; p<0·0001). When distance from the hospital, age, education level, and sex were also adjusted for, the estimated effect was similar (adjusted OR 8·27 [95% CI 3·77–18·1]; p<0·0001).

**Figure 4:**
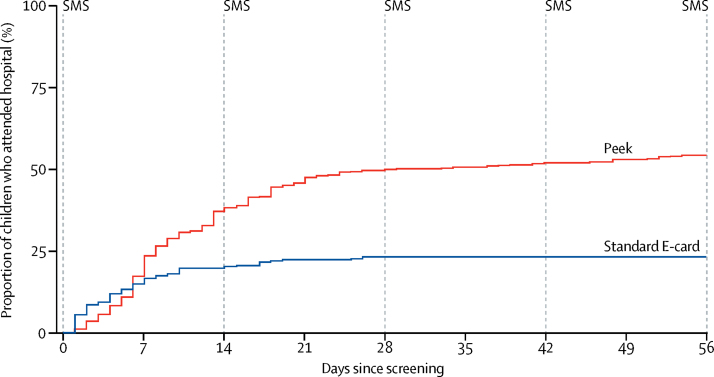
Kaplan-Meier analysis of time from screening to attendance at the hospital ophthalmology clinic SMS=short message service.

The rate of hospital attendance among those who screened positive for visual impairment was significantly higher in the Peek group than the standard group (HR 2·56 [95% CI 1·43–4·56]; p=0·0001; figure 4; table 4). However, because hazards were not proportional (p<0·0001), the time was split into weekly sections and HR was estimated for each week (table 4). This HR estimation was only possible for the first 4 weeks of follow-up because after this time no children arrived to hospital from the standard group. We did not find an intervention effect in week 1 (HR 1·03 [95% CI 0·54–1·98]; p=0·92). However, in week 2, evidence suggests that children referred using Peek had an increased attendance rate, with an estimated HR of 4·63 (95% CI 2·15–9·95; p=0·0001). Stronger intervention effects were seen in weeks 3 (HR 5·01 [95% CI 2·00–12·52]; p=0·0006) and 4 (HR 11·51 [2·41–54·93]; p=0·002).

**Table 4:** Children who attended hospital after initial referral during each week of the trial

	**Peek group**	**Standard group**	**Hazard ratio (95% CI)**	**p value**
Week 1	91 (17%)	54 (15%)	1·03 (0·54–1·98)	0·9232
Week 2	105 (37%)	17 (19%)	4·63 (2·15–9·95)	0·0001
Week 3	46 (46%)	9 (22%)	5·01 (2·00–12·52)	0·0006
Week 4	20 (49%)	2 (22%)	11·51 (2·41–54·93)	0·0022
Week 5	5 (50%)	0 (22%)	..	..
Week 6	6 (51%)	0 (22%)	..	..
Week 7	6 (53%)	0 (22%)	..	..
Week 8	6 (54%)	0 (22%)	..	..

Data are number of children (cumulative %), unless otherwise specified.

Of the children referred from schools in the standard group, 37 (47%) of 78 children were confirmed to have visual impairment (four had missing visual acuity data) compared with 68 (25%) of 276 children referred from schools in the Peek group (nine had missing visual acuity data; table 5). A higher proportion of false positives were identified among children screened using Peek than among those screened using the standard screening (p<0·0001). However, the absolute number of confirmed visually impaired children was higher in the Peek group (n=68) than the standard group (n=37). Most of the children referred who were not found to have visual impairment in the clinic had a diagnosis of allergic conjunctivitis (139 [67%] of 208 children in the Peek group and 32 [78%] of 41 children in the standard group; table 5). All children who had visually significant refractive error (<6/12) were offered free spectacles and three children had cataract surgery.

**Table 5:** Visual acuity status and diagnosis of children who screened positive for visual impairment who then attended the hospital

	**Peek group**	**Standard group**
**Visual acuity among children attending hospital**		
Children in each group	276*	78†
6/12 or better in both eyes	208 (75%)	41 (52%)
Worse than 6/12 in either eye (visual impairment confirmed)	68 (25%)	37 (47%)
**Visual acuity in the worst seeing eye of children without visual impairment in hospital**		
Children in each group	208	41
6/5	3 (1%)	0
6/6	107 (51%)	23 (56%)
6/9	66 (32%)	13 (32%)
6/12	32 (15%)	5 (12%)
**Diagnosis among children without visual impairment in hospital**		
Children in each group	208	41
Normal eyes	7 (3%)	1 (2%)
Allergic conjunctivitis, including vernal kerato-conjunctivitis	139 (67%)	32 (78%)
Refractive error	21 (10%)	4 (10%)
Others	5 (2%)	0
Not stated	36 (17%)	4 (10%)
**Visual acuity in the worst seeing eye of children with visual impairment (in either eye) in hospital**		
Children in each group	68	37
6/18	19 (28%)	14 (38%)
6/24	13 (19%)	7 (19%)
6/36	8 (12%)	4 (11%)
6/60	9 (13%)	3 (8%)
5/60 or worse	19 (28%)	9 (24%)
**Diagnosis among children with visual impairment (in either eye) in hospital**		
Children in each group	68	37
Allergic conjunctivitis, including vernal kerato-conjunctivitis	6 (9%)	3 (8%)
Refractive error	31 (46%)	26 (70%)
Corneal scars	4 (6%)	2 (5%)
Globe abnormalities	9 (13%)	3 (8%)
Cataracts	2 (3%)	1 (3%)
Others	8 (12%)	1 (3%)
Not stated	8 (12%)	1 (3%)

Data are n (%).

* Vision from nine children was not recorded.

† Vision from four children was not recorded.

## Discussion

Early identification and management of visual impairment in children is important to enable participation in education and society.^10^ We showed that an integrated system comprising a smartphone-based visual acuity test (Peek Acuity), a printed referral card illustrating the degree of visual impairment, and SMS reminders (ie, the Peek school eye health system) significantly improved the overall hospital attendance rate among children referred compared with the standard system. In this first trial, to assess the use of smartphones for vision screening and referral, we found the test can be effectively delivered by school teachers.

The rate of hospital attendance was initially similar in both groups. However, in the standard group attendance slowed after the first week before stopping completely after 4 weeks. The initial similar attendance in both groups might have been due to early responders who seek medical attention faster. Hospital attendance was better maintained in the Peek group. As the Peek system is an intervention package involving both repeated SMS and a special referral card illustrating visual impairment, which elements led to the increased attendance is unknown. The reminder messages appeared to have no additional effect on attendance after the first two reminders were sent (figure 4).

In the validation study, we found the sensitivity of Peek and the standard E-cards in detecting visual acuity of less than 6/12 to be about 75% when used by school teachers compared with about 100% for ETDRS LogMAR chart used by a clinician. Most of the false negative individuals had an EDTRS LogMAR visual acuity close to the threshold level. The negative predictive values of both tests were very high.

The specificity and positive predictive value were lower for Peek than the standard system, resulting in more children being referred who were not subsequently found to have visual impairment. However, many of them were noted to have an ocular condition. A low positive predictive value could overburden the health system with unnecessary referrals and costs, resulting in increased pressure on limited eye-care services.^12^ These false positive results might have arisen for a number of reasons: subtle variation in the smartphone screen angle, reflections off the screen or variation, and increased glare from a bright screen in the presence of inflammatory eye conditions, such as allergic conjunctivitis.^23^

To reduce the false positive rate, we propose additional testing strategies. This involves retesting the vision of all children who initially screened positive. A referral is only triggered if the child fails to meet the threshold acuity on the repeat test. If a child fails the first test and then passes the second test, a third screening test is delivered (maximum three tests per eye). Referral is triggered on confirmation of two of three failed tests. An alternative approach, currently being tested, involves extension of the number of optotypes shown to confirm the acuity level. Additionally, a set number of children who pass the screening test will be prompted by the examiner to deliver a repeat test to enable monitoring of false negative rates.

This trial suggests that, for every 10 000 children screened with standard methods, 80 of those are expected to be referred to attend the hospital clinic—38 with visual impairment and 42 without. With the Peek system, 269 children are expected to attend hospital—66 with visual impairment and 203 without. Therefore, with use of the first iteration of the Peek school eye health system an anticipated additional 28 visually impaired children will present to the clinic for assessment and treatment for every 10 000 children screened. This comes at a cost of an extra 161 children without visual impairment presenting on the basis of the methods used in this trial.

Measurements of visual acuity in children attending hospital were done with a Snellen chart several days or weeks after their initial assessment; therefore, visual acuity could have fluctuated, accounting for some of the differences. Short-term to medium-term test-retest variation in visual acuity has been reported previously.^24^, ^25^ Visual acuity is usually delivered as a continuous test from large to small angles of resolution. However, decisions for referral are made based on a threshold from that continuous test—eg, <6/12. For practical reasons, given the volume of children being screened and the need for a referral decision rather than an acuity score being the primary driver, a threshold acuity test is appropriate for screening. Most acuity tests have a one line tolerance (ie, limits of agreement) and thus delivering a threshold test is likely to result in under or over referrals of those whose true acuity falls above or below the threshold.

Of note is that most of these false positives for visual impairment were found to have some ocular pathology, most frequently allergic eye disease, which is particularly common in this population. The risk of overburdening the health system might be reduced by the delivery of triage services in or close to the school to review all children who screened positive and to manage minor eye ailments, and, where capacity allows, the assessment and delivery of refractive services referring only those who require further hospital-based treatment onwards to secondary care. A direct-to-hospital or additional triage step both require balancing outreach service capacity with health service demands for that population.

A major limitation of the current system is the low specificity of the threshold testing algorithm. In our previous study in adults,^26^ we found a substantially higher specificity for severe visual impairment using a full visual acuity as opposed to a threshold acuity testing algorithm, suggesting that modifications to the testing algorithm could improve this result. A two-staged Peek school eye health system that provides screening in the school and triage services delivered in or close to the school could optimise the benefits of its use while minimising the potential overload of the health system. This system has subsequently been refined based on findings from this trial and is being deployed to support comprehensive child eye health services to all public primary schools in Trans Nzoia County (n=340) in partnership with the Ministries of Health and Education. The triage system for refractive services recommendation has been developed into an iteration of the system that was successfully deployed in Botswana and is now being prepared for a nationwide scale-up. Further research is needed to systematically assess the barriers to accessing child eye health services and to develop and test contextually relevant measures to improve on these barriers as shown in the Peek school eye health system trial in progress in India.^27^

In conclusion, the Peek school eye health system resulted in a substantial increase in the proportion of children who attended the hospital clinic for assessment after screening positive for visual impairment and provided real-time visibility to the health system. This outcome indicates the potential value of this technology in improving uptake of services and encouraging improvement in delivery through identification of areas with potential bottlenecks in the care pathway (such as regions with the highest number of children who have not attended the hospital). The Peek Acuity screening algorithm used in this trial was less specific than the Tumbling-E card in identifying children with visual impairment. Additionally, ongoing work is required to further refine the testing algorithm, maintaining sensitivity while improving specificity without substantially increasing the testing time and systematically reducing barriers to patient care across the entire patient care pathway.
