# Correction to *Lancet Glob Health* 2018; 6: e924–32

**DOI:** 10.1016/S2214-109X(18)30358-9

**Published:** 2018-07-30

*Rono HK, Bastawrous A, Macleod D, et al. Smartphone-based screening for visual impairment in Kenyan school children: a cluster randomised controlled trial.* Lancet Glob Health *2018; **6**: e924–32*—One of the author's names has been corrected to “Gian Luca Di Tanna”. This correction has been made as of July 30, 2018.

